# Microinstability of the hip—it does exist: etiology, diagnosis and treatment

**DOI:** 10.1093/jhps/hnv017

**Published:** 2015-04-20

**Authors:** Michael M. Kalisvaart, Marc R. Safran

**Affiliations:** Division of Sports Medicine, Department of Orthopaedic Surgery, Stanford University

## Abstract

Symptomatic hip microinstability is now recognized as a potential cause of pain and disability in young patients. Causes of hip microinstability include underlying bony or soft tissue abnormalities and iatrogenic injuries of the hip capsule; however, many patients lack a clear underlying etiology. Treatment usually begins with an extensive course of non-operative management with an emphasis on activity modification and physical therapy. Surgical intervention should focus on treatment of the underlying cause as well as any associated intra-articular pathology. In many cases, arthroscopic suture plication can be considered when bony deficiency is not the cause. In this article, we will review the spectrum of symptomatic hip microinstability with a focus on the relevant anatomy, etiology, diagnosis and various treatment options.

## INTRODUCTION

Hip instability is generally defined as extraphysiologic hip motion that causes pain with or without symptoms of hip joint unsteadiness [1]. The diagnosis and treatment of hip dislocation and subluxation as a result of trauma has been well described. Symptomatic hip microinstability, however, has not received as much attention, as it is more poorly defined, has a less dramatic clinical presentation, lacks consistent objective evaluative criteria, and it has only recently emerged as a significant cause of pain and disability in younger patients and athletes. The proposed pathomechanism of hip microinstability begins with subtle anatomic abnormalities in the presence of repetitive hip joint rotation and axial loading as seen in sports such as golf, figure skating, gymnastics, ballet, martial arts, football, tennis and baseball [1, 2]. Alternatively, it may be the result of inherent ligamentous laxity and/or peri-articular muscular weakness. This results in increased movement of the femoral head relative to the acetabulum and eventual damage to the labrum, cartilage and capsular structures. Hip microinstability is often seen in patients with underlying bony abnormalities or connective tissue disorders; however, many patients with hip microinstability lack a clear underlying etiology. As a result, the diagnosis of hip microinstability is based on a thorough patient history, physical exam and radiographic evaluation. As with the shoulder, atraumatic hip instability should initially be treated with rehabilitation and strengthening of the muscles about the hip. The surgical treatment of hip microinstability should focus on addressing the underlying etiology and associated intra-articular pathology. If there is no significant bony deformity of the hip joint, treatment often consists of arthroscopic capsulorrhaphy or plication of the capsuloligamentous structures. Usually, there is associated labral pathology that should also be addressed, as the labrum is an important stabilizer of the hip [1, 2].

## ANATOMY

The hip joint has traditionally been modeled as a highly constrained concentric ball and socket joint. However, several recent anatomic and finite elemental analysis studies have demonstrated that the relationship between the femoral head and acetabulum is actually not perfectly congruent or spherical [3–6]. Under physiologic loads, there can be flattening and widening of the weight-bearing surface and as much as 2-5 mm of translation of the hip joint center [4–8].

Normal hip stability is dependent on the relationship between the bony and soft tissue structures. The acetabulum is a quasi-hemispheric structure that covers ∼170° of the femoral head [9]. This coverage is increased by the soft tissue labrum which is in circumferential continuity with the bony acetabular rim. The width of the labrum is usually between 3 and 8 mm and results in an increase in acetabular surface area by ∼25% and an increase in acetabular volume by ∼20% [10]. The acetabulum is oriented in the pelvis in an anteverted position, with ∼15-20° of anterior tilt and 45° of lateral tilt [11]. The proximal femur has ∼130° of superior inclination from the shaft (neck-shaft angle) and is in ∼10° of anteversion. This combination of femoral and acetabular offset, lateral inclination and anteversion results in more posterior bony coverage and inherent stability, allowing for more hip flexion and abduction than extension and adduction. As a result, there is also greater reliance on soft tissue structures for anterior stability, especially with the hip in an extended, adducted and externally rotated position.

The soft tissue structures of the hip joint consist of the acetabular labrum, ligamentum teres and the capsuloligamentous complex. As previously mentioned, the intact labrum is in continuity with the bony acetabular rim and increases both the surface area and volume of the socket. This results in increased hip stability and distribution of joint stresses during loading. In addition, the labrum acts as a seal between the central and peripheral compartments of the hip joint, thereby maintaining negative intra-articular pressure and creating a suction effect. In a recent biomechanical study, it was noted that 60% less force was required to distract the hip joint in the presence of a labral tear, supporting the concept of the importance of the labrum in hip stability [12, 13]. The ligamentum teres is pyramidal and somewhat flattened soft tissue structure that originates from the transverse acetabular ligament and posterior inferior acetabular fossa and inserts into the femoral head at the fovea capitis. The ligamentum tightens in a position of hip adduction, flexion and external rotation and it has been suggested that it plays a role in hip stability [1, 14, 15].

**Fig. 1.**
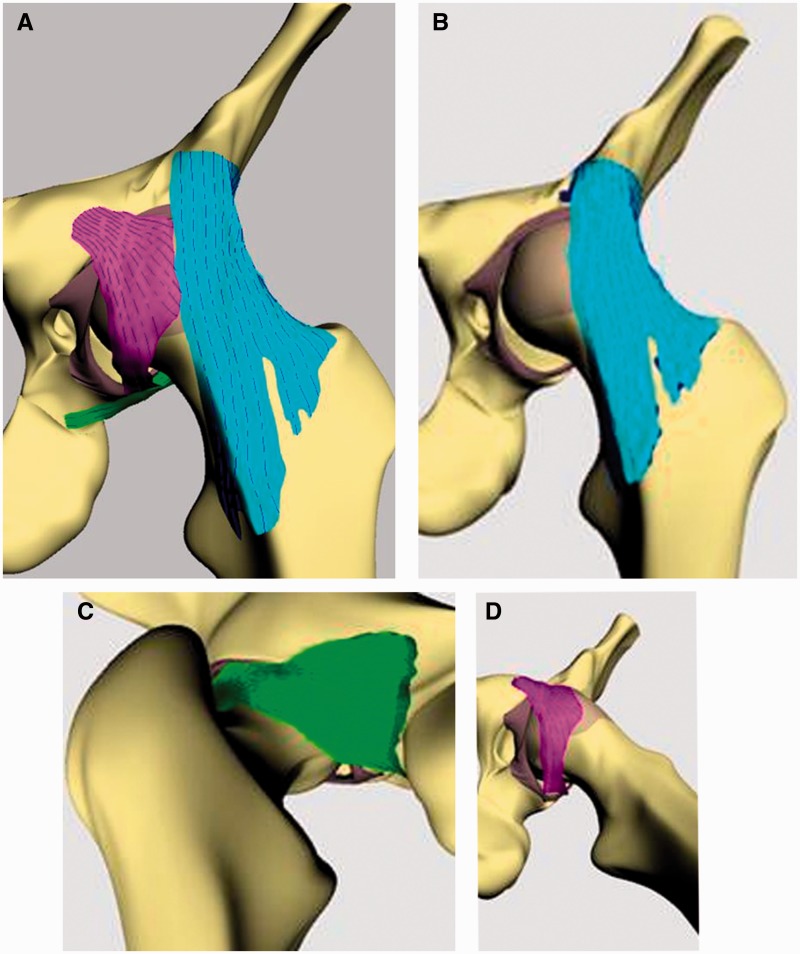
(**A**) The three longitudinal ligaments of the hip joint form a helical spiral structure around the proximal femur. (**B**) ILFL. (**C**) PFL. (**D**) ISFL.

The hip capsuloligamentous complex consists of four intimately related structures—the iliofemoral ligament (ILFL), pubofemoral ligament (PFL) and ischiofemoral ligament (ISFL) and the circular zona orbicularis [16, 17]. The first three longitudinal ligamentous structures spiral around the femoral head and insert on to the acetabulum just proximal to the labrum, creating a recess between the capsuloligamentous structures and labrum (Fig. 1). The ILFL is the strongest of the named ligaments and is also known as the Y ligament of Bigelow. It has an inverted Y shape with a single proximal attachment at the base of the anterior inferior iliac spine. It then splits into two distinct arms as it travels distally—the lateral arm crosses the joint obliquely and inserts on the anterior prominence of the greater trochanter while the medial arm travels inferiorly and inserts on the anterior femur at the level of the lesser trochanter. The ILFL limits external rotation in hip flexion and both internal and external rotation in hip extension. The PFL originates on the anterior acetabulum and travels inferiorly and posteriorly, wrapping around the femoral head like a sling or hammock. The PFL does not have a bony distal insertion; instead it blends with the medial arm of the ILFL and distal ISFL. The PFL limits external rotation, especially in hip extension. The ISFL originates on the ischial acetabular margin and spirals superolaterally as a single band to insert at the base of the greater trochanter. The ISFL limits internal rotation in both flexion and extension as well as limits posterior translation. The zona orbicularis is an intra-articular band of fibers that encircles the femoral neck. It constitutes the narrowest area within the hip capsule and has been shown to be important in limiting axial distraction of the hip joint [18].

The helical spiral orientation of the three longitudinal ligaments around the proximal femur combined with the circular zona orbicularis creates the hip ‘screw home’ mechanism. In the potentially unstable position of hip extension, the capsuloligamentous structures tighten and compress the femoral head into the acetabulum. Conversely, these fibers untwist and loosen with hip flexion, adduction and external rotation, resulting in less soft tissue constraint in this more inherently stable position, which has the additional contribution of the ligamentum teres.

The anatomy of the capsuloligamentous structures plays an important role in hip arthroscopy, especially when discussing its role in the treatment of hip microinstability. As opposed to the intra-articular zona orbicularis, the three named longitudinal ligaments cannot be seen arthroscopically from within the joint. Their anatomy has been described in a recent cadaveric study based on a clock face system, with their margins defined by the arthroscopic portals and intra-articular landmarks [16]. The ligaments reinforce ∼60% of the hip capsule that can be visualized during arthroscopy, leaving ∼40% of the capsule uncovered (the three spaces between the three ligaments) [1, 16].

The role of the musculotendinous structures about the hip in stability of the joint has not yet been elucidated. However, peri-articular muscle contraction likely helps provide hip stability by increasing the joint reaction forces and compressing the femoral head within the acetabulum [1, 2]. Additionally, the iliopsoas musculotendinous unit may provide additional stability to resist anterior femoral head translation based on its anatomic location.

## ETIOLOGY

The proposed pathomechanism of hip microinstability begins with anatomic abnormalities of the hip, whether subtle or more significant, in the presence of repetitive forces across the joint [1, 2]. These forces usually consist of repetitive joint rotation and axial loading as seen in sports such as golf, figure skating, gymnastics, ballet, martial arts, football, American football, tennis and baseball. These repetitive forces may cause damage to the soft tissue stabilizers of the hip, including the labrum and capsuloligamentous complex. This can then result in increased translation of the femoral head relative to the acetabulum, which in turn can stress these surrounding soft tissue structures further. Ultimately, the abnormal femoral head translation can cause increased tension on the labrum as well as microtrauma to the joint capsule that can lead to labral breakdown and capsular ligament stretching resulting in symptomatic hip microinstability. This can eventually lead to damage to the bony and chondral surfaces, potentially resulting in early degenerative changes of the hip joint, as articular cartilage performs suboptimally to shear stresses. These individuals may or may not have underlying joint laxity.

When discussing the etiology and treatment of hip microinstability, it is useful to divide patients into six categories based on underlying cause—significant bony abnormalities or developmental dysplasia of the hip (DDH), connective tissue disorders, post-traumatic, athletics/microtrauma, iatrogenic and idiopathic.

DDH is present in ∼1% of the western population and represents a spectrum of hip pathology ranging from mild hip dysplasia to severe bony deformity resulting in hip subluxation and dislocation [19]. Typical anatomic changes in DDH include a misshapen femoral head, a shallow acetabulum with loss of anterolateral coverage, increased acetabular lateral tilt and excessive anteversion of the acetabulum and proximal femur [20]. The combination of these bony abnormalities can result in anterior hip instability and early degenerative changes due to these abnormal hip joint forces. Although DDH is a problem of femoral head undercoverage, overcoverage of the femoral head resulting in femoroacetabular impingement (FAI) is also thought to be a potential etiology of hip microinstability. Cam impingement occurs when excessive bone at the femoral head-neck junction collides with the acetabular rim, resulting in articular cartilage delamination and damage. Pincer impingement occurs when there is excessive bone along the acetabular rim as a result of conditions, such as acetabular retroversion or coxa profunda, resulting in labral damage and tears. In terms of hip instability, it is thought that impingement of the femoral head and neck against the acetabular rim in the extremes of hip range of motion can result in levering of the head out of the socket. In addition, a significantly retroverted acetabulum may even result in symptomatic posterior instability due to a lack of posterior coverage. This may then be accentuated by the anterior acetabular overcoverage, allowing for levering of the femoral head-neck region.

Patients with connective tissue disorders, such as Ehlers-Danlos syndrome, Marfan syndrome or Down syndrome, may also be predisposed to instability of the hip and other joints. Abnormal collagen formation and content can result in laxity of the hip capsuloligamentous structures, resulting in hip instability and subluxation in positions with less inherent bony stability.

Post-traumatic microinstability occurs when dislocation or subluxation of the hip secondary to a traumatic event, such as a motor vehicle crash or sports injury, results in damage to the capsuloligamentous structures about the hip, with or without associated tears of the labrum [21]. The most common mechanism of hip dislocation is posterior, caused by a posteriorly directed force transmitted through a bent knee, such as landing on a bent knee or being tackled with the hip and knee flexed [1]. Hip dislocations without associated fracture are felt to be inherently stable; therefore, immediate surgical stabilization is usually not warranted. In some patients, however, there may be residual laxity of the hip joint as a result of the extensive soft tissue damage, resulting in symptomatic microinstability.

Repetitive microtrauma to the hip capsuloligamentous structures may also result in symptomatic microinstability. As previously mentioned, sports requiring repetitive hip joint rotation and axial loading may place athletes at risk for repetitive microtrauma to the hip joint. In addition, some athletes may have subclinical laxity of their soft tissues which allows them to achieve extremes of hip range of motion. This can be advantageous in athletes participating in sports such as dance, gymnastics and swimming (including synchronized swimming)—similar to the shoulder in a throwing athlete—but also may place them at a higher risk for hip instability and injury.

Iatrogenic hip instability is relatively uncommon, but has been reported in the literature. The majority of cases of symptomatic hip instability occur following total hip arthroplasty, but it may also present after open hip procedures such as surgical hip dislocations requiring trochanteric osteotomy and capsulotomy [22]. In addition, there have been several reports in the literature of hip instability and even dislocation following hip arthroscopy in which a capsulotomy/capsulectomy was performed without closure [23–26].

Finally, patients with idiopathic hip microinstability lack significant bony abnormalities, a diagnosis of an underlying connective tissue disorder, or history of previous trauma or hip surgery. Additionally, they may have any or several associated factors: mild generalized ligamentous laxity (or a subclinical connective tissue disorder), mild hip dysplasia not meeting radiographic diagnosis, mild FAI or other osseous abnormalities, and/or focal capsular redundancy and laxity [1].

## DIAGNOSIS

The diagnosis of hip microinstability as a result of significant bony abnormalities or an underlying connective tissue disorder is relatively straightforward due to the clear underlying etiology. The diagnosis of idiopathic hip microinstability, however, can be much more challenging as the presentation may be quite subtle. There is no definitive preoperative diagnostic test, physical exam finding or imaging modality that can be used to definitively diagnose idiopathic hip microinstability. Instead, the clinician must have a strong suspicion for hip microinstability based on the overall clinical evaluation. This includes the patient history, physical exam and available radiographic imaging. If there is a strong suspicion for hip microinstability, examination under anesthesia can be of significant value.

Most patients with idiopathic hip microinstability have a chief complaint of hip pain, although some will note apprehension or a sense of giving way during certain activities. Special attention should be given to symptoms brought on by activities requiring repetitive hip rotation and axial loading, such as those seen in sports noted earlier. The nature of the onset of symptoms should also be noted—most patients with idiopathic microinstability experience an insidious onset and gradually worsening of symptoms without a specific history of trauma or a specific precipitating event. However, a history of traumatic onset does not rule out hip microinstability, as it may be the factor that makes a susceptible athlete become symptomatic. Any previous ipsilateral hip injuries or surgeries should also be noted, as instability could be iatrogenic in nature or previously undiagnosed and untreated. In a study from 2007, the authors noted that 35% of patients undergoing revision hip arthroscopy required capsulorrhaphy at the time of revision, suggesting that undiagnosed hip microinstability may have contributed to the need for revision surgery [27]. In a recent study, describing nine patients requiring revision hip arthroscopy, 78% of the included patients had radiographic evidence of capsular and iliofemoral defects on magnetic resonance arthrography (MRA) [28].

A thorough physical exam is extremely important when the diagnosis of hip microinstability is suspected. The goal during the physical exam should be to reproduce the patient’s symptoms, whether pain or apprehension, with range of motion, palpation and/or provocative tests. Pain as a result of intra-articular hip pathology is generally localized to the groin, buttock, thigh, or in the ‘C sign’ distribution and usually cannot be reproduced with direct palpation [29, 30]. Hip strength and range of motion should be evaluated, and special attention should be made to the spine, abdomen and knee to rule-out associated pathology and/or a referred source of symptoms. Excessive internal or external rotation of the hip (>60° in either direction), and/or lateral knee joint line <3 inches from the examination table with the leg in a figure of 4 position may be suggestive of increased laxity of the hip joint. The presence of generalized ligamentous laxity/hypermobility should be assessed using the Beighton criteria—small finger passive dorsiflexion >90°, thumb passive dorsiflexion to the flexor aspect of the forearm, elbow hyperextension >10°, knee hyperextension >10° and palms flat on the floor with forward flexion and knees fully extended [31]. If there is concern for an underlying connective tissue disorder, appropriate referral for medical and/or genetic evaluation may be warranted.

**Fig. 2.**
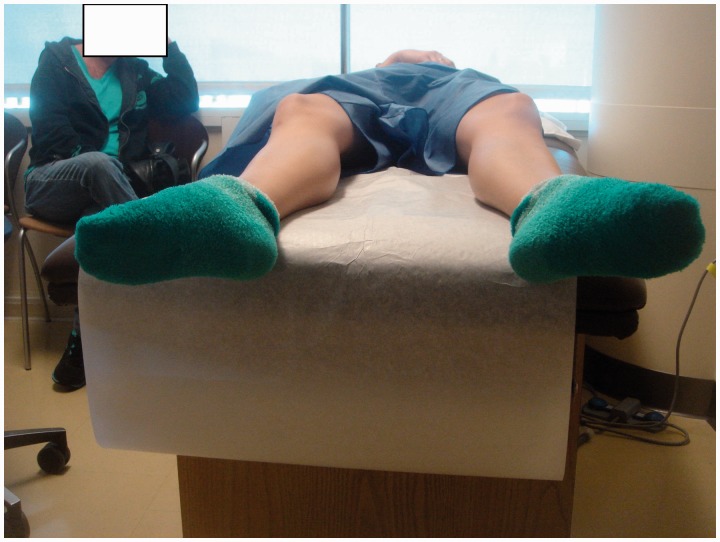
Log roll. As the patient is laying supine, the resting position of her right leg is significantly more externally rotated than her left leg, consistent with laxity of her ILFL. Generally, this test starts with the patient supine, and the examiner rotates the leg internally and then lets the leg passively fall into external rotation.

**Fig. 3.**
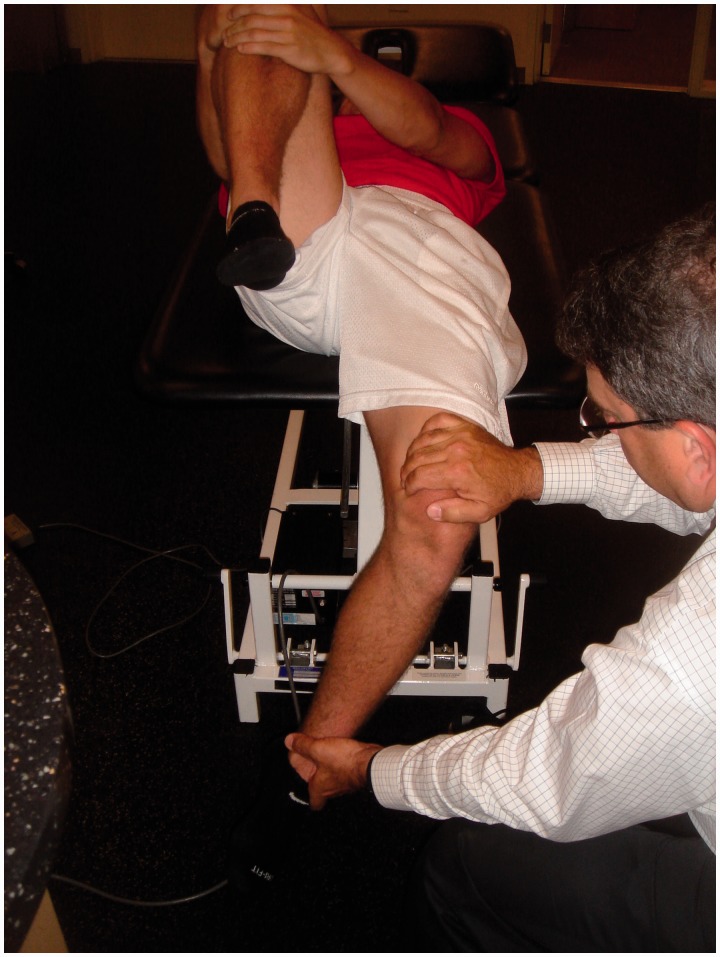
Anterior apprehension test. Also known as the hyperextension—external rotation test. The subject is positioned at the end of the examination table. The patient holds one knee with the hip in flexion, while the other hip is allowed to extend over the end of the table. The examiner then externally rotates the extremity. Anterior hip pain is consistent with anterior labral tear and/or anterior hip instability. Posterior hip pain may be present with posterior hip impingement.

**Fig. 4.**
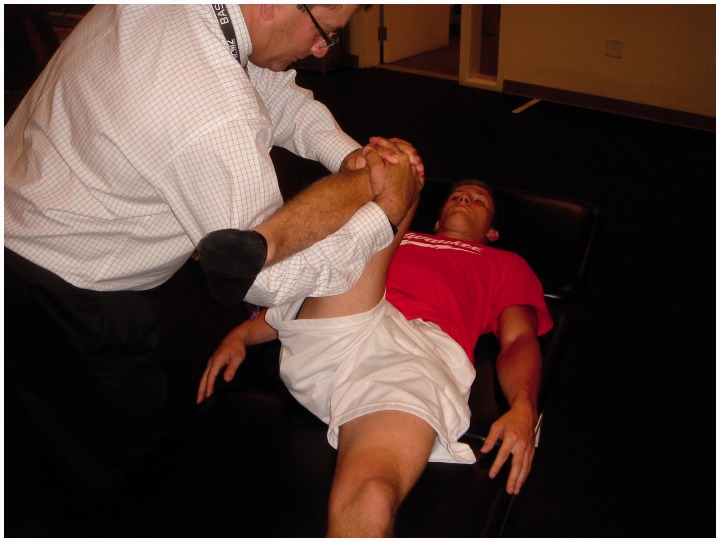
Posterior apprehension test. With the patient supine, the examiner flexes the hip to 90°, adducts, internally rotates and then applies a posterior force on the knee. This will cause posterior pain or sensation of instability.

**Fig. 5.**
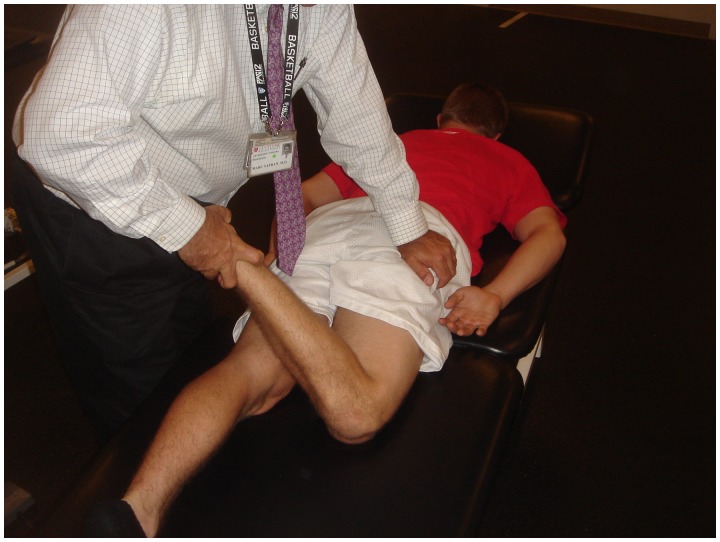
Prone external rotation test. The patient is prone for this test. The affected hip is maximally externally rotated with anteriorly directed pressure on the posterior greater trochanter to translate the femoral head anteriorly. A positive test reproduces the patient’s symptoms in this position.

**Fig. 6.**
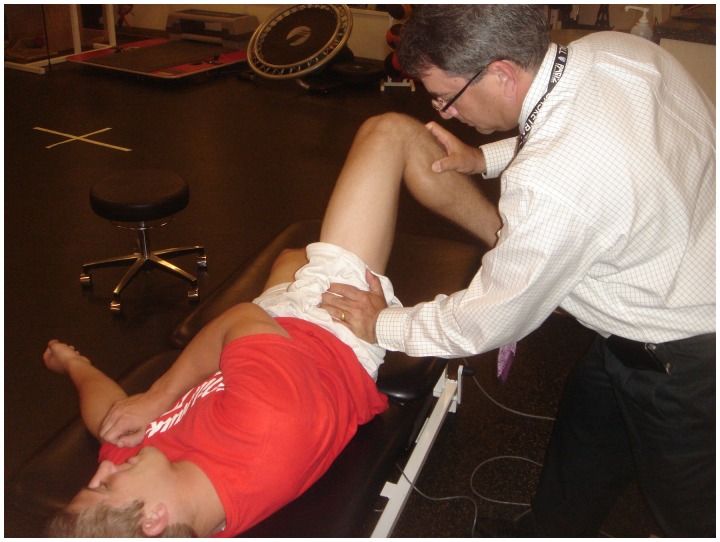
Abduction-extension-external rotation test. With this test of anterior instability, the patient is placed in the lateral decubitus position with the affected side up, abducted to 30° and externally rotated. An anteriorly directed pressure is placed on the posterior greater trochanter and the leg is slowly extended from 10° of flexion to full extension with a positive test reproducing the patient’s symptoms.

The anterior impingement test can be used to diagnose intra-articular hip joint pain (including FAI or acetabular retroversion), and the labral stress test can be used to detect the presence of a labral tear [32]. In addition, five specific provocative maneuvers have been described to evaluate for hip instability—the log roll test, anterior apprehension test, posterior apprehension test, prone external rotation test and abduction-extension-external rotation test. The log roll test is performed with the patient in the supine position [1]. With the patient relaxed, the examiner internally rotates the foot past neutral (straight up) and removes pressure from the foot. The foot will then fall back into external rotation. External rotation greater than the contralateral side may suggest anterior capsular laxity (especially if the foot-table angle is <20°) and can be considered a positive test (Fig. 2). The anterior apprehension test is performed with the patient in the supine position with the buttocks just to the edge of the examination table—the affected lower extremity is then extended (although the patient holds the contralateral extremity in flexion) and externally rotated [1]. The maneuver stresses the anterior hip capsule, and a positive test reproduces anterior hip pain and/or apprehension (Fig. 3). The posterior apprehension test is performed with the patient in the supine position with the affected hip in 90° of flexion, adduction and internal rotation [1]. A posteriorly directed force is then applied and a positive test reproduces pain and/or apprehension (Fig. 4). The prone external rotation test, described by Domb, is performed with the patient prone and the affected hip maximally externally rotated with anteriorly directed pressure on the posterior greater trochanter to translate the femoral head anteriorly [33]. A positive test reproduces the patient’s symptoms in this position (Fig. 5). Finally, the abduction-extension-external rotation test is performed with the patient in the lateral position with the affected side up, and the hip abducted to 30° and externally rotated [34]. Pressure is placed on the posterior aspect of the greater trochanter and the leg is slowly extended from 10° of flexion to full extension while applying an anteriorly directed force through the greater trochanter, with a positive test reproducing the patient’s symptoms (Fig. 6).

**Fig. 7.**
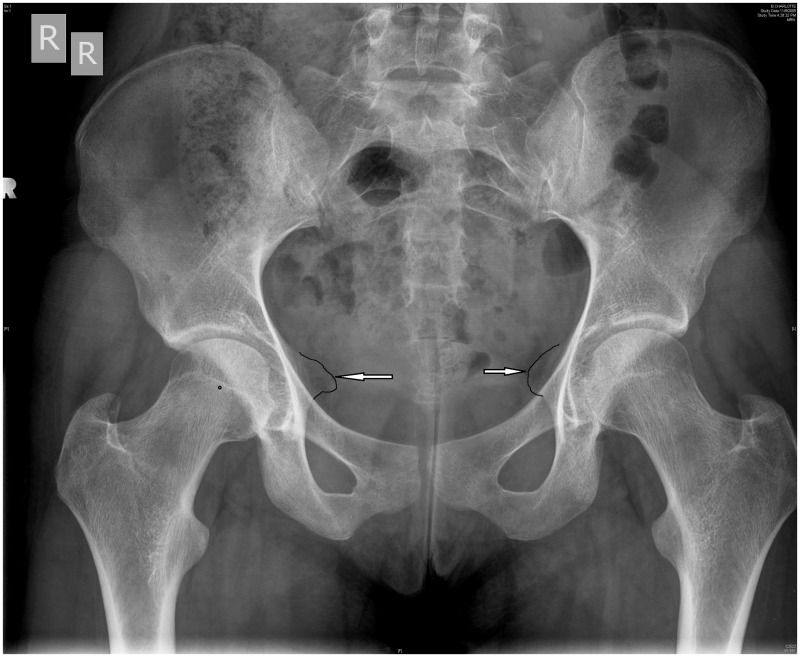
AP radiographs of a female volleyball player with right hip pain, and a history of right hip posterior dislocation. The small circle is in the center of the femoral head. Clearly, the posterior wall of the acetabulum is medial to the center of the femoral head—this is called the posterior wall sign. This demonstrates posterior wall insufficiency. The black line outlines the ischial spines with the arrows pointing to the prominent ischial spines. This would suggest the patient has acetabular retroversion, which is the reason for her posterior wall insufficiency.

Plain film radiographs should include high-quality supine anteroposterior (AP) pelvis and cross-table lateral views. Radiographs should be inspected for dysplasia, FAI, previous trauma and degenerative changes. Acetabular dysplasia can be defined as a lateral center-edge angle of Wiberg of <20-25° on the AP pelvis. A Tonnis angle (angle of acetabular inclination) >10° is also suggestive of acetabular dysplasia. Acetabular retroversion is suggested by the posterior wall sign and/or ischial spine sign. The posterior wall sign is present when the posterior wall of the acetabulum is medial to the center of the femoral head on the AP pelvis view (Fig. 7). This suggests posterior wall insufficiency, possibly due to a retroverted acetabulum. The ischial spine sign is present when the projection of the ischial spine extends into the pelvis on the AP pelvis view; once again suggesting possible retroversion of the acetabulum (Fig. 7). Anterior undercoverage, measured by the anterior center edge angle, can be assessed with a false profile view of Lequensne.

**Fig. 8.**
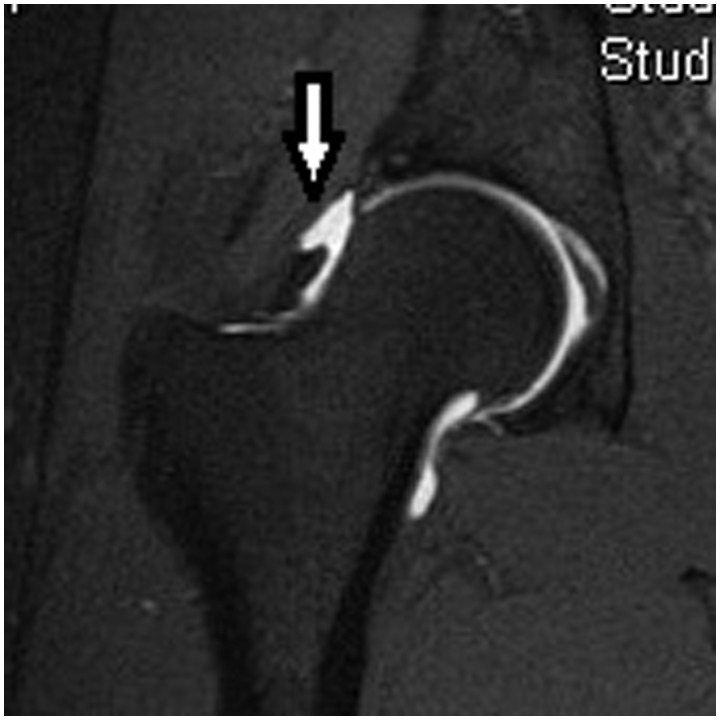
MR arthrogram, coronal view, of a patient with symptomatic hip instability following a hip arthroscopy. Note the defect in the capsule (arrow).

Magnetic resonance imaging is often performed to better evaluate the intra-articular structures, such as the labrum, joint cartilage and capsuloligamentous structures. MRA with intra-articular gadolinium has been shown to better assess cartilage and soft tissue pathology [35]. MRA can be especially useful in cases of possible hip microinstability, as the intra-articular contrast distends the hip capsule and can demonstrate capsular redundancy or iatrogenic injury (Fig. 8). Magerkurth *et al.* [36] recently retrospectively reviewed preoperative MRA imaging in patients with hip joint capsular laxity noted at the time of arthroscopy. They found that hip joint laxity was associated a widened anterior hip joint recess (>5 mm) and a thinned adjacent joint capsule (<3 mm) lateral to the zona orbicularis. It should be noted that this was a retrospective study performed at a single center, and further research is required to validate these findings.

The physical exam is not always reliable in predicting intra-articular hip pathology and multiple studies have also demonstrated that labral tears are often present in asymptomatic individuals [37–39]. As a result, a diagnostic intra-articular injection of local anesthetic is performed on every patient with suspected intra-articular hip pathology at our institution. The injection is performed under image guidance (fluoroscopic or ultrasound) and is performed either with the MRA or in isolation. The patient is asked to record the percentage of pain relief within the first few hours following the injection. If the patient’s pain is related to intra-articular pathology, the majority of their symptoms should dramatically improve following the injection. Persistent or minimal improvement in symptoms following the injection should prompt either a repeat injection to confirm that the injection is intra-articular or further workup to search for other potential sources of pain.

**Fig. 9.**
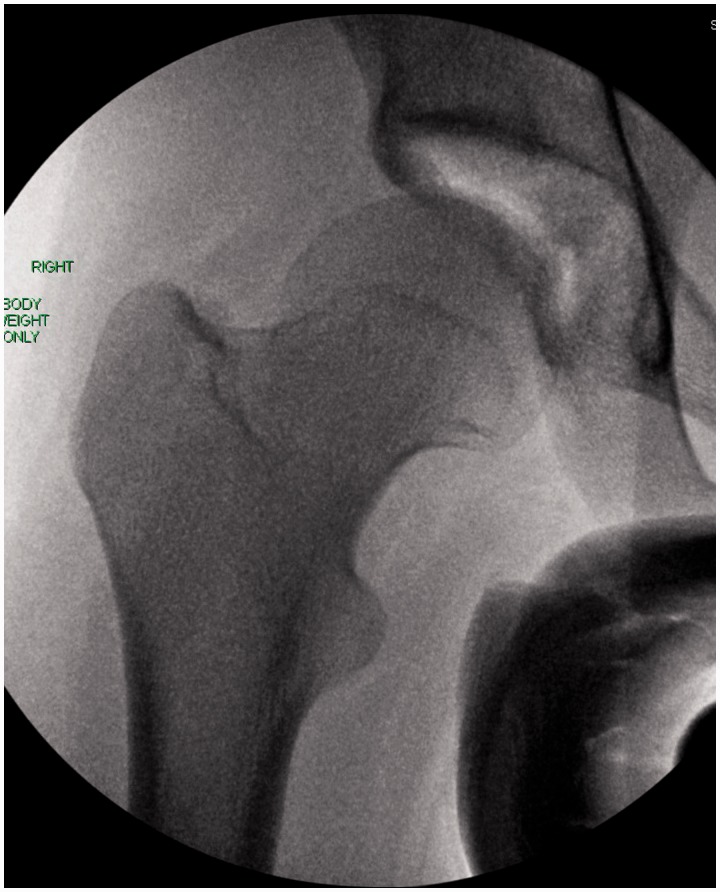
Intra-operative fluoroscopic image of a patient where just manual traction (using body weight, but no traction applied using fracture table) resulted in femoral head distraction of a few millimeters. This is consistent with hip instability. This patient had a capsular plication and labral repair, with complete resolution of her symptoms.

**Fig. 10.**
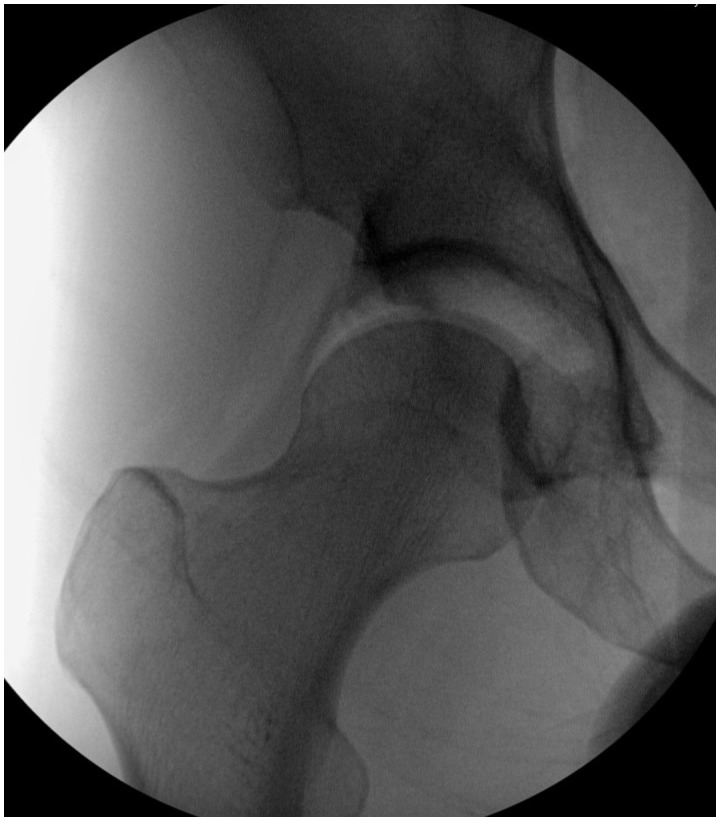
Same patient as shown in Fig. 9, though after a spinal needle was introduced to remove negative intra-articular pressure, and the traction force was removed, her femoral head remained displaced, also consistent with hip instability.

If there is a strong suspicion for hip instability based on the patient history, physical exam, radiographic evaluation and diagnostic intra-articular injection, an examination under anesthesia is often very useful to confirm hip joint laxity. Examination under anesthesia is usually performed at the time of hip arthroscopy, and a number of different techniques have been described. Dynamic fluoroscopy can be used to evaluate hip stability in positions of pain or apprehension previously noted on physical exam [1, 14]. A traction view of the affected hip can sometimes demonstrate the ‘vacuum’ sign, indicating abnormal distraction across the hip joint [40–42]. In our practice, hip joint laxity is assessed under fluoroscopic guidance with the use of manual traction and the number of ‘turns’ on the traction table at the time of hip arthroscopy. Easy hip distraction or subluxation with manual distraction suggests significant hip instability (Fig. 9). In addition, we also note the number of ‘turns’ of traction required to distract the joint 7-10 mm is noted—with our current fracture table, less than 10 ‘turns’ suggests hip instability and ligamentous laxity. Once traction is released, we again assess the hip joint with fluoroscopy. A femoral head that remains unreduced or lateralized relative to the acetabulum with no traction applied, after removal of negative intra-articular pressure, also suggests laxity of the capsuloligamentous structures (Fig. 10).

Intra-articular damage frequently seen in patients with hip microinstability include labral tears directly anteriorly or directly laterally, inside-out chondral wear at the acetabular rime and central femoral head chondral lesions.

## TREATMENT

The treatment algorithm of hip microinstability depends on the severity and frequency of symptoms as well as the underlying cause. In cases of significant bony deformity such as severe acetabular dysplasia or acetabular retroversion, open redirectional osteotomies of the acetabulum and/or proximal femur may be required. In addition, patients with gross instability of the hip (frank subluxation or dislocation) would warrant early operative intervention. In general, however, the treatment for patients with hip pain and evidence of microinstability usually begins with an extensive course of non-operative management. This includes activity modification, oral anti-inflammatory medications and a course of formal physical therapy with a focus on strengthening of the iliopsoas, hip abductors, short external rotators, abdominal core muscles and low back. In patients with an underlying connective tissue disorder, we also often recommend an extended course of non-operative management as a result of their abnormal collagen biology. There are no reports in the literature on the outcomes of non-operative treatment of hip microinstability, but it has been our anecdotal experience that a significant number of patients improve without surgery. In addition, there have been reports in the literature demonstrating clinical improvement in patients with intra-articular pathology treated with non-operative management [43]. If there has been no improvement in symptoms after a dedicated 8–12 week course of non-operative treatment after 8–12 weeks, surgical intervention may be considered.

In patients with symptomatic hip instability who have failed non-operative modalities, surgical soft tissue balancing should be considered. As previously mentioned, cases of severe bony deformity such as severe acetabular dysplasia or retroversion may require open redirectional osteotomies. In the absence of significant abnormal bony anatomy, however, treatment options should focus on the hip capsuloligamentous complex. Several techniques have been described to shrink or reduce hip capsular volume, either via open or arthroscopic approaches [9, 14, 33, 44]. Several authors have successfully treated atraumatic hip instability with open capsulorrhaphy [42, 45–47]. The advantages of an open technique are excellent visualization, ease of access to the joint and the ability to address associated bony deformity. Advances in hip arthroscopy over the last several years, however, have now allowed surgeons to address hip microinstability in a less invasive manner. There are no studies in the literature that directly compare open and arthroscopic procedures, but it is generally felt that arthroscopic techniques should be considered as first line treatment when capsular redundancy, labral tears and/or bony abnormalities are within the working area of the arthroscope [1].

When treating hip microinstability via hip arthroscopy, it is important to address associated intra-articular pathology and consider the condition of the hip capsule. The labrum is known to be important for hip stability and cartilage protection. As a result, labral tears should be repaired if possible rather than simply debrided and/or removed. For patients with labral deficiency or irreparable labral tears in the context of hip instability, labral reconstruction should be considered. Capsulotomies created during access to the hip joint should be made as small as possible in patients with capsular laxity, and closure should also be strongly considered. As previously mentioned, there have been reported cases of hip dislocation following arthroscopic capsulotomy/capsulectomy without closure [23–26]. As a result, it is recommended that any patient undergoing hip arthroscopy with capsular redundancy and/or symptomatic capsular laxity be considered for capsule repair and/or plication. In addition, capsular repair and/or plication should be strongly considered in patient with generalized ligamentous laxity or an underlying connective tissue disorder that are undergoing arthroscopic treatment of labral tears, cartilage damage or FAI [48]. For those patients who have capsular defects, open or arthroscopic capsular reconstruction should be considered if the defect cannot be closed primarily.

Arthroscopic techniques to treat hip capsular redundancy and reduce capsular volume include thermal capsulorrhaphy and suture plication. Arthroscopic thermal capsulorrhaphy of the hip was first reported by Philippon [9]. The hip capsular volume is reduced with the use of thermal energy by laser or radiofrequency; at a temperature of 65–70°C, there is collagen degeneration and subsequent tissue shrinkage. Thermal capsulorrhaphy in the shoulder has been associated with thermal necrosis, chondrolysis and capsular attenuation, although these complications have not been reported in the hip [49, 50]. Regardless, concern for these potential complications has led to arthroscopic hip capsular plication with suture becoming more commonly used for treating hip capsular laxity and microinstability. Although more technically demanding, this technique allows control of plication tension and can easily be used during concomitant cartilage, labrum or FAI procedures. Several authors have reported success with a variety of arthroscopic suture plication techniques; however, most of these reports consist of small case series [24, 27, 51]. Larson *et al.* [48] recently presented the outcomes of suture plication with labral surgery, in addition to 12 rim resections and 12 osteochondroplasties, in 16 hips of patients with Ehlers-Danlos syndrome. The average improvement in the modified Harris hip score was 43 points, with 90% of the patients reporting good to excellent results at an average of 40 months after surgery. The technique utilized at our institution consists of arthroscopic suture plication through the capsular ‘bare area’ between the ILFL anteriorly and ISFL posteriorly.

### Surgical technique

We perform hip arthroscopy with the patient in the supine position on a traction table with a well-padded perineal post. Initial manual traction is applied to the operative extremity, while gentle counter traction is applied through the non-operative extremity. An assessment of hip joint laxity is performed under fluoroscopic visualization as previously described. Access to the joint is gained through a standard anterolateral portal followed by modified anterior and posterolateral portals placed under direct visualization.

The central compartment is initially inspected for chondral injuries, labral tears and chondrolabral delamination. A capsulotomy between portals, particularly the anterior and anterolateral portals, is not routinely performed in an attempt to maintain the integrity of the hip capsular ligaments, especially in cases of symptomatic hip instability where there is already a concern for an inadequate capsuloligamentous complex. Intra-articular pathology is addressed and an acetabuloplasty/rim resection can be performed in cases of pincer FAI. Traction is then release and attention is turned to the peripheral compartment. The femoral head-neck junction is assessed and a cheilectomy/femoral neck osteoplasty is performed in cases of cam FAI.

**Fig. 11.**
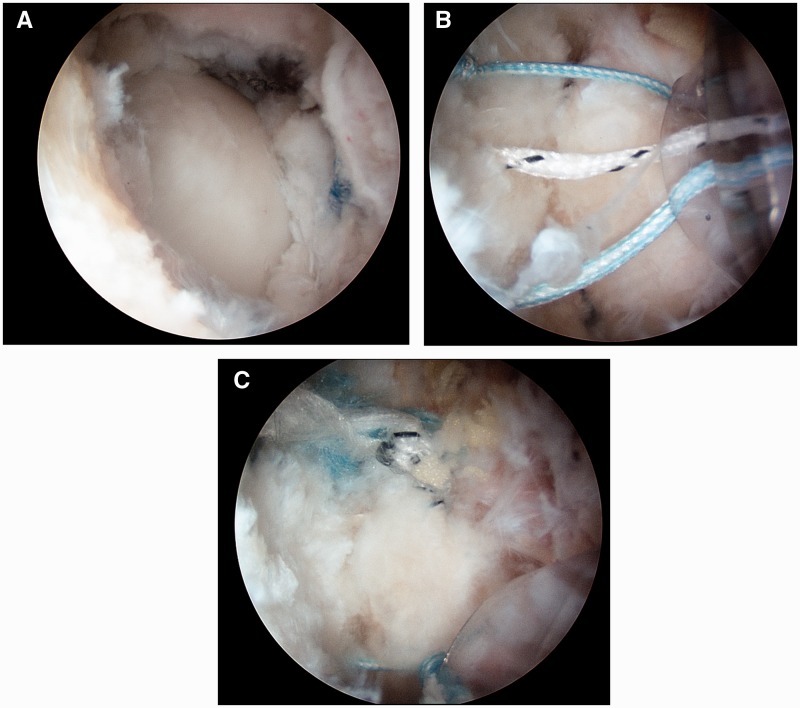
Arthroscopic photographs of the capsular plication technique. (**A**) The anterolateral partial capsulectomy is performed in the ‘bare area’ until the femoral head is visualized. (**B**) Three to five sutures are passed across the partial capsulectomy using a suture shuttling device. (**C**) The sutures are tied to perform the capsulorrhaphy.

Capsular plication is performed with a suture shuttling technique developed by the senior author. After traction is released, a proximal anterolateral portal is created 3-4 cm proximal to the anterolateral portal. A 30° arthroscopic lens is placed in the anterolateral portal, and a shaver is placed in the proximal anterolateral portal, using fluoroscopy to assist with localization. An anterolateral partial capsulectomy ∼8 mm in width (proximal-distal) and 15 mm in length (anterior-posterior) is then performed in the ‘bare area’ between the ILFL and ISFL until the femoral head is clearly visualized [16]. These sizes are approximate and vary based on the degree of patient laxity as well as on patient size. A suture shuttling technique using a curved suture shuttling device is then utilized to perform the capsulorrhaphy, essentially closing the ligament ‘bare area’ with three to five sutures depending on the size of the defect (Fig. 11). Post-operatively, patients remain limited weight-bearing in an abduction brace with range of motion limited from 0 to 90° of hip flexion for a period of 2 weeks.

Although good results have been reported with a variety of arthroscopic suture plication techniques, the technique used by the senior author is unique in that it avoids iatrogenic injury to the ILFL. The ILFL is the strongest and thickest of the capsular ligaments and its integrity is critical for resisting anterior translation of the femoral head and for stabilizing the ‘screw home’ mechanism during terminal hip extension and external rotation [1]. Several described plication techniques involve transection of the ILFL, either longitudinally during the vertical limb of a T-capsulotomy or transversely in an attempt to perform an inferior capsular shift [33, 44, 52]. When suture plication is then performed, there is a potential for over-tightening of the ILFL. This may result in hip stiffness and loss of range of motion. Domb et al. noted a mean of 11° loss of external rotation in patients treated with ILFL plication. The consequence of this loss of motion is unclear; however, in athletes participating in activities requiring extremes of motion, such as ballet dancers, this may be significant. The plication technique performed by the senior author, however, consists of a capsulotomy and plication in the capsular ‘bare area’ between the ILFL and ISFL [16]. The senior author terms this capsular ‘bare area’ as the ‘rotator interval of the hip’. Plication of this ‘rotator interval’ theoretically avoids over-tightening of a single capsular ligament. Given that there is no quantitative way to measure hip instability or a consensus on how much capsular plication is necessary to treat instability, we feel that it is safer to close this capsular interval rather than directly tightening a specific capsular ligament.

In a recent review of our outcomes of isolated arthroscopic suture plication (no concomitant bony resections of the acetabulum or proximal femur) in 32 consecutive patients with this technique for isolated hip microinstability, we noted significant improvement in patient pain and function at a minimum of 1 year follow-up [53]. In addition, no patients complained of post-operative subjective hip stiffness, and we found no significant difference between preoperative and post-operative hip range of motion.

## FUTURE

As we go forth, there is a need for further study of hip instability. The physical examination for hip instability is becoming clearer. However, there is a need for an objective measure of hip laxity, as well as hip instability. This is important for the diagnosis, as well as for possibly identifying which patients may do well with non-operative management, and which would require surgery. Furthermore, an objective measure would be helpful to assist in developing consistent techniques for treatment of hip instability. Likely, the situation may be similar with the shoulder, in that there are varying degrees of laxity in asymptomatic patients, and instability is just symptomatic laxity. However, there is no way to know until there is an objective measure that we can use to study hip microinstability.

## SUMMARY

Symptomatic hip microinstability is recognized as a potential cause of pain and disability in young patients. The etiology of hip microinstability includes bony abnormalities, residual laxity after traumatic dislocation, connective tissue disorders resulting in ligamentous laxity, repetitive microtrauma associated with athletic activities, iatrogenic injuries to the hip capsule and idiopathic. In most cases, initial treatment should consist of non-operative management, focusing on strengthening of the hip and core muscles. Surgical intervention is considered in patients with persistent symptoms despite a dedicated course of activity modification and physical therapy. Surgery should focus on treatment of the underlying cause as well as consider associated pathology, such as labral tears, capsular insufficiency and FAI. In patients with severe anatomic bony abnormalities, open redirectional osteotomies may be required. In the non-dysplastic patient, however, arthroscopic suture plication is generally considered.

## CONFLICT OF INTEREST STATEMENT

None declared.
